# PMEPA1 induces EMT via a non‐canonical TGF‐β signalling in colorectal cancer

**DOI:** 10.1111/jcmm.14261

**Published:** 2019-03-19

**Authors:** Lei Zhang, Xue Wang, Chong Lai, Honghe Zhang, Maode Lai

**Affiliations:** ^1^ School of Basic Medical Sciences and Clinical Pharmacy China Pharmaceutical University Nanjing China; ^2^ Department of Urology, School of Medicine The First Affiliated Hospital, Zhejiang University Hangzhou China; ^3^ Department of Pathology, Key Laboratory of Disease Proteomics of Zhejiang Province, School of Medicine Zhejiang University Hangzhou China

**Keywords:** CRC, EMT, PMEPA1, TGFβ

## Abstract

Prostate transmembrane protein androgen induced 1 (PMEPA1) has been reported to promote cancer progression. Metastasis is the main factor leading to cancer progression and poor prognosis, and at the beginning of metastasis, epithelial‐to‐mesenchymal transition (EMT) is a crucial activation. However, the relationship between PMEPA1 and EMT in colorectal cancer metastasis is still poorly understood. In this study, we first testified that PMEPA1 expresses higher in tumour than normal tissue in Gene Expression Omnibus database, in the Cancer Genome Atlas (TCGA) as well as in the clinical data we collected. Moreover, the higher expression was associated with poor prognosis. We furthermore demonstrated PMEPA1 promotes colorectal cancer metastasis and EMT in vivo and in vitro. We found that PMEPA1 activates the bone morphogenetic proteins (BMP) signalling of TGF‐β signalling resulting in promoting EMT and accelerating the proliferation and metastasis of colorectal cancer.

## 1 INTRODUCTION

Colorectal cancer (CRC) is one of the most common solid carcinomas all over the world. More than one million patients have been diagnosed with CRC per year, and the mortality of CRC is the third amongst all cancers.^1^ Metastasis is responsible for cancer poor prognosis, and epithelial‐to‐mesenchymal transition (EMT) is a significant program at the beginning of cancer metastasis.^2^, ^3^, ^4^ EMT is activated by several pathways, such as MAPK/ERK1/2, PI3K/AKT, FAK/Src, WNT/β‐catenin and TGF‐β/Smad pathways.^5^

The TGFβ family includes Transforming growth factor‐β (TGF‐β), activins, bone morphogenetic proteins (BMP) and growth differentiate factor (GDF). The TGF‐β pathway maintains the homeostasis and suppresses tumourigenesis at the early stage of tumourigenesis. However, with the progression of tumours, suppressive effects of TGF‐β have been circumvented and tumour cells take advantage of TGFβ family to acquire a mesenchymal phenotype.^6^, ^7^ As for EMT, it is demonstrated that EMT is activated via the TGFβ/Smad‐dependent pathway in several cell lines. After binding with TGF‐β1, TGF‐β1 receptor leads Smad2 and Smad3 to phosphorylate. The phosphorylated Smad2 and Smad3 then combine with Smad4 to form a complex, which is translocated to the nucleus and promote the transcription of target genes, for example, the EMT related genes. Meanwhile, the Smad2/3/4‐independent pathway also activates EMT.^8^

Prostate transmembrane protein, androgen induced 1, PMEPA1 (also called TMEPA1, STAG1, ERG1.2 or N4WBP4), is located at chromosome 20q13. PMEPA1 expresses at the membrane of the cell and some subcellular organelles, such as endoplasmic reticulum and Golgi apparatus. PMEPA1 contains a transmembrane domain at the N‐terminus and two PY motifs (PPxY) which interact with WW domain of the E3 ubiquitin ligase Nedd4. PMEPA1 was firstly found in prostate cancer as an androgen‐induced gene.^9^ However, PMEPA1 has duplicate roles in prostate cancer. In androgen receptor‐positive prostate cancer cell, PMEPA1 promotes the proliferation. But in the androgen receptor‐negative prostate cancer cells, PMEPA1 inhibits the proliferation by suppressing Smad3/4–c‐Myc–p21Cip1.^10^ PMEPA1 also inhibits proliferation through the inhibition of NEDD and PTEN in prostate cancer.^11^, ^12^ Moreover, PMEPA1 inhibits the bone metastasis by blocking the TGF‐β and androgen signalling in prostate cancer.^13^ In ovarian cancer, PMEPA1 has been reported to promote apoptosis.^14^ However, PMEPA1 promotes the cancer progression in other solid cancer,^15^ such as lung cancer,^16^, ^17^ breast cancer,^18^, ^19^, ^20^ gastric cancer^21^ and ovarian cancer.^22^ However, the anticancer role of PMEPA1 has been reported to promote apoptosis.^14^ The difference might be resulted from tissue‐specificity. Different types of cancers exhibit different and special characteristic. As for PMEPA1, some studies have shown PMEAP1 inhibited proliferation through androgen receptor,^11^ however, androgen receptor and the related signalling pathways have been activated in prostate cancer, but maybe not in lung cancer or breast, or colorectal cancer.

And the different role of PMEPA1 can be explained by the relationship of PMEPA1 and TGF‐β pathways. PMEPA1 is induced by the TGF‐β signalling, but meanwhile, it inhibits the phosphorylation of Smad2 and Smad3 to antagonize TGF‐β signalling.^23^ Considering the negative loop of PMEPA1 and TGF‐β signalling, the phenotype of PMEPA1 in specific cancer needs deeper investigation.

PMEPA1 in CRC is associated with cancer poor prognosis. Moreover, we built stable PMEPA1 overexpressed and PMEPA1‐knockdown CRC cell lines to demonstrate that PMEPA1 promotes the cancer cell proliferation via inhibiting G1/S cell cycle arrest and inducing EMT related tumour metastasis. Besides, we investigated the regulation of PMEPA1 on TGF‐β signalling. PMEPA1 blocked the canonical TGF‐β signalling via dephosphorylating of Smad2 and Smad3. Interestingly, the BMP signalling, a non‐canonical TGF‐β signalling also promoted EMT and the pathway was activated by PMEAP1 which was BMP‐depended in CRC. These new findings have thrown light on the role of PMEPA1 in colorectal cancer.

## 2 MATERIALS AND METHODS

### 2.1 Public datasets analysis

CRC expression profiling studies including relevant clinical information were identified by searching the public datasets. Dataset GDS2947 included 32 prospectively collected adenomas with those of normal mucosa from the same individuals.^24^ And the comparison between 40 paired colorectal adenoma and adjacent normal tissue samples were performed by dataset GSE31737.^25^ Datasets with gene expression profile comparing CRC or colorectal adenoma to paired adjacent normal tissue were obtained from Dataset GSE32323 which contained 17 paired samples.^26^ GSE41328 contained five colorectal adenocarcinomas and matched normal colonic tissues were analysed with Affymetrix HG‐U133‐Plus‐2.0 microarrays.^27^ GSE38832 includes survival information of 122 patients with CRC.^28^ GSE17537 includes expression and clinical data for 55 patients with CRC.^29^ CRC expression and copy number profiling study from TCGA dataset were used to analysis association and survival.

### 2.2 Pathway enrichment analysis

The correlated genes with PMEPA1 were screened in TCGA database and GSE35834 dataset by Pearson product‐moment correlation analysis. A threshold of *P *< 0.05 and odds ratio >0.3 were used to screen the gene with significant correlations. The GSEA tool (http://software.broadinstitute.org/gsea/msigdb/) was used for pathways enrichment.

### 2.3 Clinical specimens

This study was approved by the ethics committee of Zhejiang University's School of Medicine and was carried out in accordance with the approved guidelines and regulations. One hundred and fifty‐five CRC patients were recruited from Sir Run Run Shaw Hospital of Zhejiang University. Pathologic diagnoses were evaluated by pathologists via biopsy reports. Patients with familial adenomatous polyposis, hereditary non‐polyposis CRC and inflammatory bowel disease were excluded. All tissue samples were obtained from colorectal adenocarcinoma patients without any adjuvant treatment including radiotherapy or chemotherapy prior to surgery and diagnosis.

### 2.4 Cell lines and cell culture

The human colon cancer cell lines SW620, HT29, HCT116, HCT 8 and HEK293t were purchased from the American Type Culture Collection (Manassas, VA, USA), and all the colon cancer cell above were cultured in RPMI 1640 medium (HyClone, Tauranga, New Zealand) with 10% foetal bovine serum (HyClone, Tauranga, New Zealand). HEK293T cell was cultured in DMEM high Glucose (HyClone, Tauranga, New Zealand) with foetal bovine serum (HyClone, Tauranga, New Zealand). All the cells are grown at 37℃ in an atmosphere of 95% and 5% CO_2_.

### 2.5 DNA and siRNA constructs

The full CDS sequence of PMEPA1 was amplified and cloned into p3×FLAG‐CMV‐14 (Sigma). The pLKO.1 lentivirus vector was used to construct shRNA‐PMEPA1 vector, and lentiviruses were co‐transfected into HEK293T cells with the packaging plasmids pMD2.G and psPAX2. Lipofectamine 2000 (Invitrogen) was used in all transfection experiments according to its manufacture instructions. And the siRNA‐Negative (UUCUCCGAACGUGUCACGUTT), siRNA‐PMEPA1(GGAGCUGGAGUUUGUUCAGTT) and siRNA‐smad1(CCAAUAGCAGUUACCCAAATT) were transfected by GenMute siRNA Transfection Reagent (SignaGen).

### 2.6 Stable cell lines

All transfections were performed with Lipofectamine 2000 (Invitrogen, USA) according to the manufacturer's instructions. HCT8 and HCT116 cell lines expressing PMEPA1 stably were obtained by transfection with pCMV‐3 × flag vector containing PMEPA1 DNA and selected in 10μg/ml G418 for 2 weeks. SW620 and HT29 PMEPA1 knocked‐down cell lines were built by the lentivirus which is produced by the HEK293T transfected with the pLKO vector containing sh‐PMEPA1 and selected in 1 μg/mL puromycin for 2 weeks.

### 2.7 Cell proliferation assay

2×10^3 ^cells were plated in 96‐well plates, and 0, 24, 48, 72 and 96 hours after plating 10 μL CCK8 solution (Boster, Wuhan, China) was added to each well. Subsequently, the cells were incubated for 3 hours at 37℃ and 5% CO_2_. The supernatants were removed to new 96‐well plates and recorded the optical absorbance at 490 mn.

### 2.8 Plate clone assay and soft agar clone assay

2×10^3^ cells were plated in 6‐well plates and fixed by 4% (w/v) paraformaldehyde and stained by 0.1% crystal violet after 2 weeks. Soft agar colony formation assay was carried out as described previously. 2×10^3 ^cells were plated on the 6‐well plates coated soft agar. Three weeks after seeding, the cell was fixed by 4% (w/v) paraformaldehyde and stained by 0.1% crystal violet.

### 2.9 Cell cycle and cell apoptosis assay

Cells were harvested and stained with the PI Cell cycle kit and Annexin V/PI Cell Apoptosis Detection Kit (MultiSciences, Hangzhou, China) according to the manufacturer's instructions. Data acquisition and analysis were performed with Bection Coulter Flow Cytometer using WinMDI.

### 2.10 Cell migration and invasion assay

The migration and invasion capacity of cells were tested by transwell migration and transwell invasion assays. The cell was plated in the upper compartment chambers of 24‐well plates equipped with cell culture inserts containing 8.0 μm pore size membrane (Costar Corp. Cambridge, MA, USA) with 1% FBS medium and the lower chamber was containing 10% FBS medium. Diluted extracellular matrix gel (BD Biosciences Bedford, MA, USA) was coated in the upper chamber for the invasion assays, but not migration assays. Moreover, 5 × 10^4^ cells were incubated for migration assays, and for the invasion, assays 1 × 10^5^ cells are required. The cells in the chamber were fixed in 4% paraformaldehyde and stained by 0.1% crystal violet 48 or 72 hours after incubating. Moreover, 30% acetic acid was used to wash the chamber, and the washing solution was recorded the optical absorbance at 570 mn.

### 2.11 Wound healing

1×10^5^ cells were plated in per wells of 6‐wells plate and 10 μL tip was used to scratch the well 24 hours after plating. The scratched monolayer cultures were photographed 0, 72 hours after starching.

### 2.12 Western blot, immunohistochemistry and immunofluorescence

Western blot, immunohistochemistry and immunofluorescence were performed as previously.^30^, ^31^ And the related antibodies were used as following: PMEPA1 (Santa Cruz Biotechnology Inc; 1:700 for WB, 1:75 for IHC), E‐cadherin (Cell Signaling Technology; 1:1000 for WB, Dako; 1:250 for IHC; Santa Cruz Biotechnology Inc 1:500 for IF), Vimentin (Cell Signaling Technology; 1:1000 for WB, 1:500 for IF; Dako, 1:4000 for IHC), MMP9 (Cell Signaling Technology; 1:1000 for WB; 1:200 for IHC), Twist (Santa Cruz Biotechnology Inc, 1:200 for WB) Snail (Cell signaling Technology, 1:1000 for WB) Smad2/3, Smad1, p‐Smad2/3, p‐Smad1/5/8 (Cell Signaling Technology, 1:1000 for WB) and GAPDH (Multi Sciences, China, 1:5000 for WB). GAPDH was used as a loading control for Western blots.

### 2.13 Co‐immunoprecipitation

Co‐IP lysis buffer (20 mmol/L Tris‐HCl, 150 mmol/L NaCl_2_, 1.5 mmol/L EDTA, 0.5 mmol/L NaVO4, 0.5% NP‐40, pH = 8.0) with complete protease inhibitor were used for harvest whole cell lysate. The cell lysates were incubated with beads overnight at 4℃. The beads (Anti‐FLAG® M2 Magnetic Beads, Sigma) were washed by washing buffer (20 mmol/L Tris‐HCl, 50 mmol/L NaCl2, 1.5mmol/L EDTA,0.5mmol/L NaVO4, 0.5% NP‐40, pH=8.0), and eluted by elution buffer (1M Tris, pH = 2.0) for the Western blot assay.

### 2.14 RNA extraction and quantitative RT‐PCR

Total RNA from the tissues were extracted using TRIZOL Reagent (Invitrogen). The RNA concentration was determined using UV spectrophotometry. cDNA was synthesized with PrimeScript ^®^ RT regent kit (Takara Biotechnology, Dalian, China). RT‐PCR was performed with Thunderbird SYBR Master Mix (Takara, Japan). The PCR was performed on a Real‐time PCR Detection System (StepOnePlus, ABI) with the following cycles: 95°C for 1 minute, followed by 40 cycles of 95°C for 15 seconds, 60°C for 15 seconds and 72°C for 45 seconds to detect the target gene level and GAPDH gene levels. GAPDH expression was used as an internal control. The 2^−ΔCT^ was calculated for every sample and normalized to GAPDH.

### 2.15 Animal experiment

Male BALB/c nude mice, 4 weeks and 18‐22 g, (supplied by Shanghai Laboratory Animal Limited Company) were used for animal study which is approved by the Animal Ethics Committee of Zhejiang University, China. 1×10^6^ Control cells and PMEPA1 overexpression or PMEPA1 silenced cells were subcutaneously inoculated per nude mice. The weight of mice and the volume of the tumour were measured twice a week. Four weeks after inoculating, the nude mice were killed and the tumours were measured and weighed. Tumour volume (TV) was calculated using the following formula: TV (mm^3^) = D/2 × d^2^, where D and d are the longest and the shortest diameters, respectively.

Male 5 weeks NOD/SCID mice were used for animal studies of tumour metastasis. The control cells and PMEPA1 knockdown cells, pGKV5‐LUC Neo vector (1 × 10^6^ cells) transfected and stably expressed, were suspended in 0.1 mL PBS and were intravenously injected into the tail vein. Post intraperitoneal injections of 1.5 mg of luciferin (Gold Biotechnology, USA) for 10 minutes, the metastases were monitored using the IVIS@ Lumina II system (Caliper Life Sciences, Hopkinton, MA, USA). On account of excessive tumour burdens, all animals were humanely sacrificed after 5 weeks. Pieces of the lung were fixed in 10% formalin before embedded in paraffin. Serial sections of the embedded specimens were stained with haematoxylin and eosin (H&E) as conventionally conducted.

### 2.16 Statistical analysis

The statistical package SPSS (version 20.0; IBM New York, NY, USA) was applied. Unpaired Student's *t* tests were used for normally distributed data and non‐parametric Mann‐Whitney *U* tests were used for non‐normally distributed data to compare central tendencies. For results in CRC tissues, Relapse‐free, metastasis‐free or overall survival were compared between high and low PMEPA1 expression groups using median gene expression value as a bifurcating point. Correlations were analysed by the Spearman coefficient test. Significance was set at *P* < 0.05. Stata software was used to be a comprehensive evaluation that associated public datasets with clinical samples.

## 3 RESULTS

### 3.1 PMEPA1 expresses higher in tumour and associated poor prognosis

**Figure 1:**
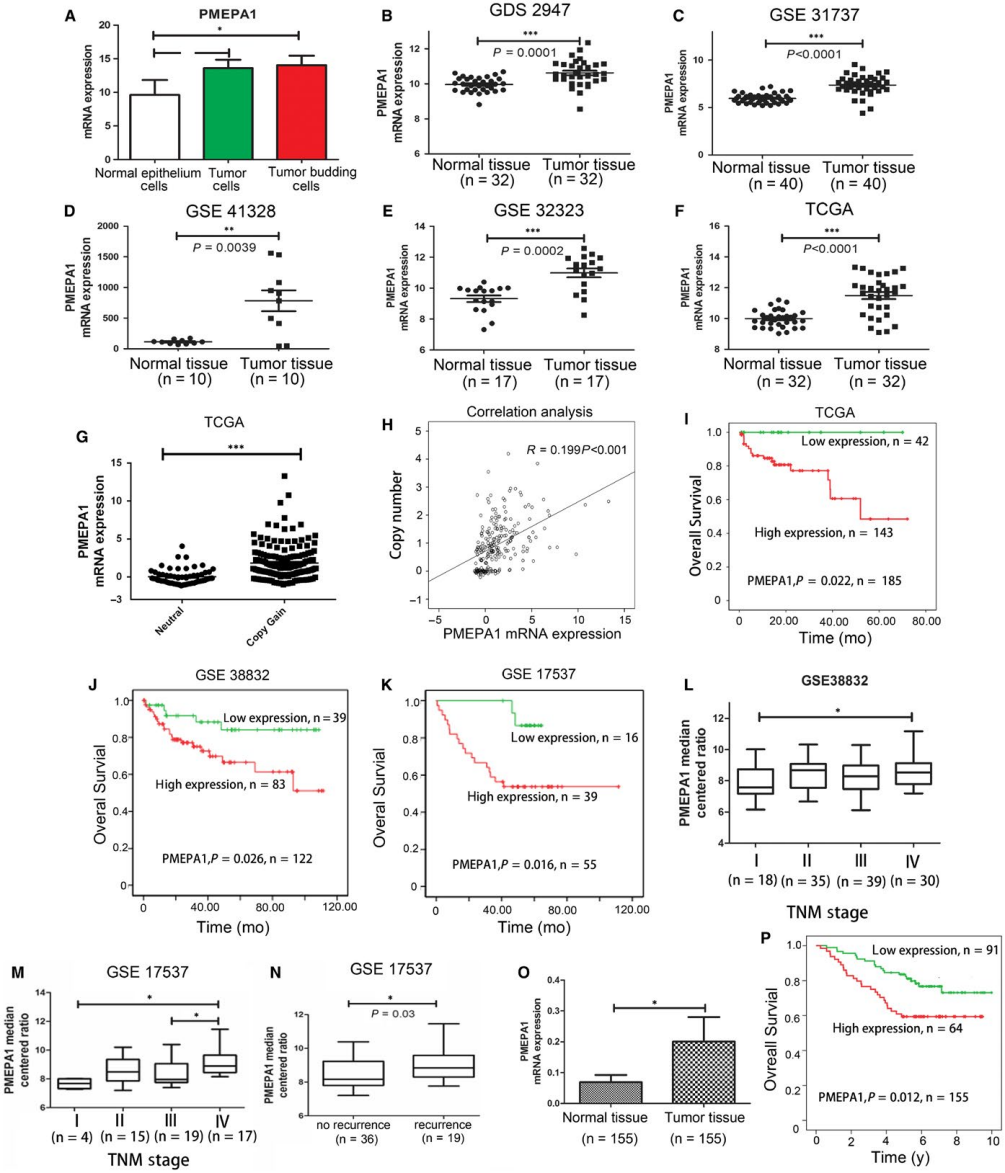
Prostate transmembrane protein androgen induced 1 (PMEPA1) expresses higher in tumour and related with poor prognosis (A) the expression of PMEPA1 in normal epithelium cells, tumour parenchyma cells and tumour budding cells in colorectal cancer. B, GSD2941 showed that PMEPA1 mRNA expressed higher in tumour tissue when compared with paired adjacent normal tissue (*P *= 0.0001). C, GES31737 showed that PMEPA1 mRNA expressed higher in tumour tissue when compared with paired adjacent normal tissue (*P* < 0.0001). D, GSE41328 showed that PMEPA1 mRNA expressed higher in tumour tissue when compared with paired adjacent normal tissue (*P* = 0.0039). E, GSE32323 showed that PMEPA1 mRNA expressed higher in tumour tissue when compared with paired adjacent normal tissue (*P* = 0.0002). F, TCGA data showed that PMEPA1 mRNA expressed higher in tumour tissue when compared with paired adjacent normal tissue (*P* = 0.0039). G, TCGA data showed that mRNA level of PMEPA1 is higher in copy number gained group (focal CNV values larger than 0.3) than neutral group (focal CNV values between and including −0.3 and 0.3). (H) The copy number and mRNA expression of PMEPA1 showed a positive correlation. (I‐K) The correlation between PMEPA1 expression and prognosis overall survival were analyzed by Kaplan‐Meier survival curve in (I) TCGA (J) GSE38832 (K) GSE17537 database. L, The relationship between PMEPA1 and TMN stage analysed by Pearson correlation analysis (*P* = 0.0023) in GSE38832. (M) The relationship between PMEPA1 and TNM stage analysed by Pearson correlation analysis (*P* < 0.05) in GSE 17537. N, The relationship between PMEPA1 and recurrence analysed by Pearson correlation analysis (*P* < 0.05) in GSE 17537. O, The relative PMEPA1 mRNA expression of normal and CRC samples from the Sun run run hospital. P, The Kaplan‐Meier survival analysis of CRC patients with high and low expression of PMEPA1. The designations for levels of significance were used within this figure: *P < 0.05; **P < 0.01; ***P < 0.001; ns, not significant

Our previous gene expression microarray and bioinformatics works have shown the PMEPA1 expresses higher in tumour cells and tumour budding cells than that in stroma cells.^32^ Tumour budding, occurring at the invasive front of cancer has a metastatic and stem‐cell‐like feature indicating a poor prognosis. Tumour budding is partly responsible for cancer metastasis, and its initiation is based on the epithelial‐mesenchymal transition (EMT) process. The expression of PMEPA1 was higher in tumour budding cells than tumour parenchyma cells and normal epithelial cells (Figure 1A). We then confirmed that mRNA expression of PMEPA1 was higher in tumour than normal tissue in TCGA and GEO database GDS2947 n = 32 *P* = 0.001 (Figure 1B), GSE31737 n = 40 *P* < 0.0001 (Figure 1C), GSE41329 n = 10 *P* = 0.0039 (Figure 1D), GSE32323 n = 17 *P* = 0.0002 (Figure 1E) and TCGA n = 32 *P* < 0.001 (Figure 1F), and all the data were from the paired samples. To identify the changes of PMEPA1 mRNA is related with copy number, we investigated the copy number of PMEPA1 in TCGA database, and found the mRNA level of PMEPA1 was increased in the copy number gained group (Figure 1G). We then analysed the relationship between copy number and mRNA expression of PMEPA1, which showed there was a significantly positive correlation between copy number and mRNA level of PMEPA1 (Figure 1H). These data indicated that mRNA level of PMEPA1 is higher in CRC tumour tissue than normal tissue.

To explore the relation between PMEPA1 and the prognosis, we obtained a validation cohort from the GEO databases. Kaplan‐Meier survival analysis was conducted to evaluate the prognostic value of the gene signature in three datasets retrieved from the GEO and TCGA database. The log‐rank test results confirmed that the PMEPA1 was closely related to overall survival in three datasets TCGA n = 185 *P* = 0.022 (Figure 1I), GSE38832, n = 122, *P* = 0.026 (Figure 1J) and GSE17536, n = 55, *P* = 0.016 (Figure 1K); and. Pearson correlation analysis confirmed that the expression of PMEPA1 was related to TNM stage in GSE38832 (Figure 1L) and GSE 17537 (Figure 1M). PMEPA1 median centred ratio was related to recurrence in GSE38832, *P* = 0.03 (Figure 1N).We also testified the samples from Sir Run Run Shaw Hospital and found mRNA level of PMEPA1 expressed higher in the tumour than normal tissue and the higher expression is related to the poor prognosis (Figure 1O,P).

Moreover, the results of TCGA database and Sir Run Run Shaw Hospital database were analysed by multivariate Cox proportional hazards regression model to find an independent prognostic value of PMEPA1 by adjusting location, differentiation, infiltrating depth, lymph node metastasis distant metastasis and TNM stage. After adjustment, PMEPA1 still showed a significant prognostic value. (Tables 1 and 2).

**Table 1:** Relationship between serum PMEPA1 level and clinical pathological characteristic of CRC patients. (TCGA database)

Characteristics	Number (n = 185)	Median expression of PMEPA1	*P* value
Age (y)			
≤60	41	0.9313	0.392
>60	144	0.5798	
Gender			
Male	98	0.3127	0.783
Female	87	0.7341	
Copy number			
Neutral	50	−0.3686	<0.0001
Gain	111	1.0874	
High level	13	1.2483	
Lymph node invasion			
N0	78	0.5678	0.076
N1	74	0.8641	
Vascular invasion			
N0	112	0.8058	0.229
N1	41	0.2931	
TNM stage			
Ⅰ	37	0.8058	0.031
Ⅱ	68	0.2009	
Ⅲ	47	0.6222	
Ⅳ	30	1.2323	

**Table 2:** Relationship between serum PMEPA1 level and clinicopathological characteristic of CRC patients. (Sir Run Run Shaw Hospital database)

Characteristics	Number (n = 155)	Median expression of PMEPA1	*P* value
Age (y)			
≤60	55	1.2259	0.237
>60	100	2.1455	
Gender			
Male	78	2.0059	0.164
Female	77	1.2692	
Location			
Colon	66	2.2302	0.203
Rectrum	89	1.4221	
Tumour size (cm)			
≤5	76	1.5166	0.637
>5	79	1.8057	
Histology			
N0	123	1.7962	0.452
N1	32	1.8244	
Differentiation			
Well	117	1.7962	0.894
Mod	13	2.3763	
Poor	24	1.7963	
Unknown	1		
Grade			
N0	130	1.8010	0.77
N1	25	1.8020	
Infiltrating depth			
N0	23	0.8256	0.027
N1	132	1.9547	
Lymph node metastasis			
N0	90	1.3728	0.057
N1	65	2.6899	
Metastasis			
N0	129	1.8057	0.818
N1	26	1.2632	
TNM stage			
Ⅰ	19	0.6624	0.029
Ⅱ	60	1.7991	
Ⅲ	50	2.7260	
Ⅳ	26	1.2632	

### 3.2 Knockdown of PMEPA1 inhibits proliferation and metastasis in colorectal cancer cells

**Figure 2:**
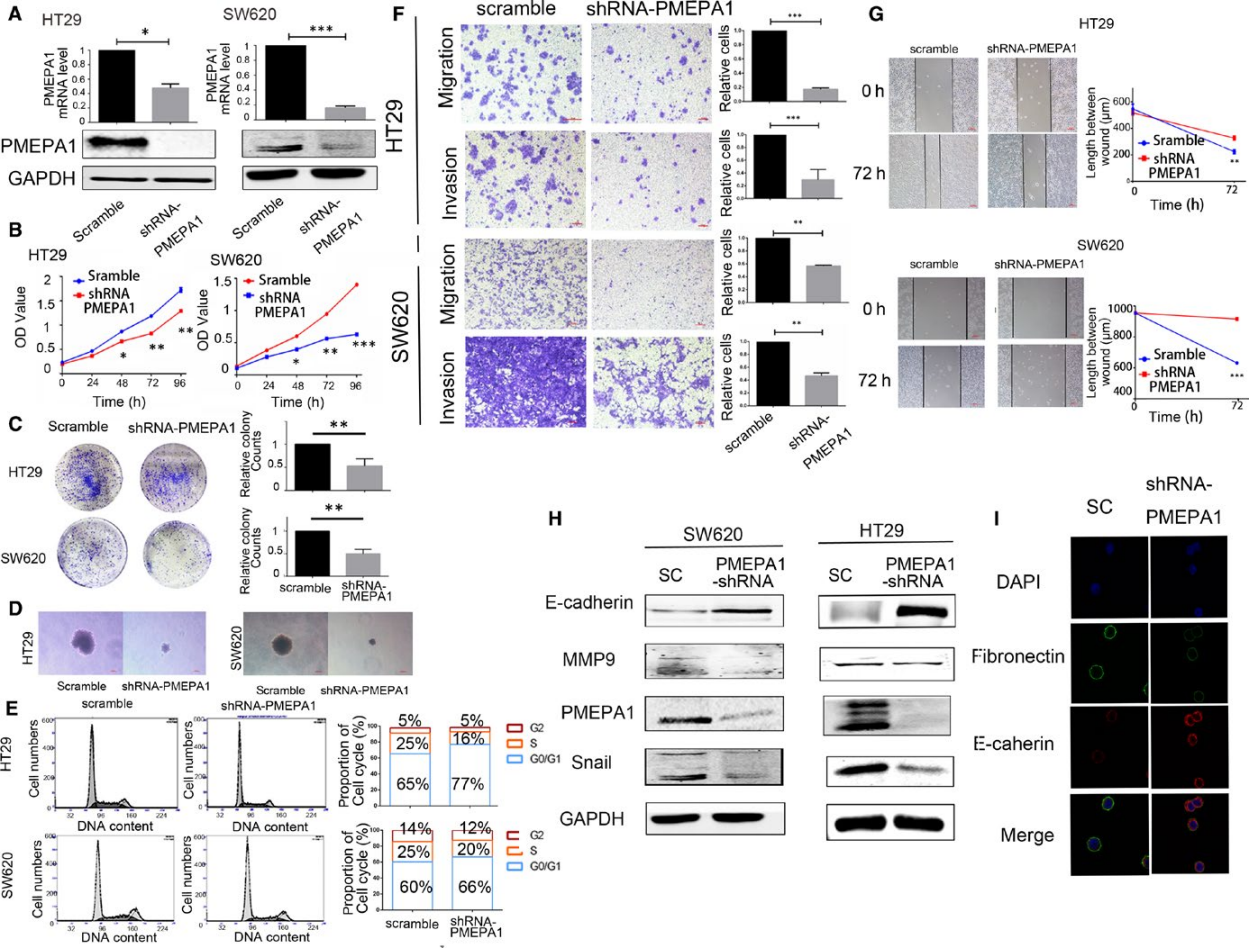
Knockdown PMEPA1 inhibits proliferation and metastasis in colorectal cancer cells. A, The efficiency of PMEPA1 knocked down has been testified by qRT‐PCR and Western blot in HT29 and SW620. (B‐D) The proliferation of HT29 and SW620 cells with PMEPA1 down‐regulation was detected by CCK8 assay, plate clone assay and soft agar clone assay. E, Flow Cytometer detected the cell cycle and the proportion of each cell cycle. F, Migration and invasion assay were used for HT29 and SW620 cells with PMEPA1 down‐regulation. The chambers were washed by 30% acetic and absorbance of washing solution was recorded at 570 mn for the quantification of the relative migration and invasion cells. G, Wound healing assay was used for the HT29 and SW620 cells with PMEPA1 down‐regulation. And the length of the wound has been measured by Image J. H, Western blot detection of E‐cadherin, MMP9, snail and PMEPA1 in HT29 and SW620 with PMEPA1 down‐regulation. I, Immunofluorescence assay for expression of E‐cadherin and Fibronectin in PMEPA1 down‐regulated SW620 cells.The designations for levels of significance were used within this figure: *P < 0.05; **P < 0.01; ***P < 0.001; ns, not significant

To investigate the molecular role of PMEPA1 in colorectal cancer cells, first, we detected the mRNA and protein level in several colorectal cancer cell lines by RT‐PCR and Western blot (Figure S1A). Subsequently, we selected PMEPA1 higher expressed cell lines, HT29 and SW620, for building the stable PMEPA1 knockdown cell lines. We then tested mRNA and protein level of PMEPA1 by RT‐PCR and Western blot, we investigated the influence on colorectal cancer cells with the comparison of relative control cell lines (Figure 2A). Compared with the Scramble‐shRNA, PMEPA1‐shRNA inhibited the proliferation and clones formation of HT29 and SW620 (Figure 2B‐D). To investigate the related mechanism of proliferation, cell cycle was analysed by flow cytometer, which showed PMEPA1 knockdown cells were arrested in the G1/S cell cycle (Figure 2E). Moreover, down‐regulation of PMEPA1 inhibited migration and invasion and reduced the capacity of wound healing (Figure 2F,G). Considering EMT is a significant process of cell migration and invasion, we detected the proteins level of EMT markers in PMEPA1 knockdown cell lines and control cell lines. As the epithelium marker, E‐cadherin was up‐regulated; as the mesenchymal markers, MMP9 and Snail were down‐regulated in the PMEPA1 knockdown cell lines, which indicates PMEPA1 knockdown inhibited EMT (Figure 2H). Immunofluorescence assay also validated that down‐regulated PMEAP1 increased expression of E‐cadherin but decreased fibronectin, a mesenchymal marker (Figure 2I). Taken together, the data shows PMEPA1 knockdown arrests cell at G1/S and inhibits CRC cell proliferation and PMEPA1 knockdown inhibits EMT and metastasis of CRC cells.

### 3.3 PMEPA1 promotes proliferation and metastasis in colorectal cancer cells

**Figure 3:**
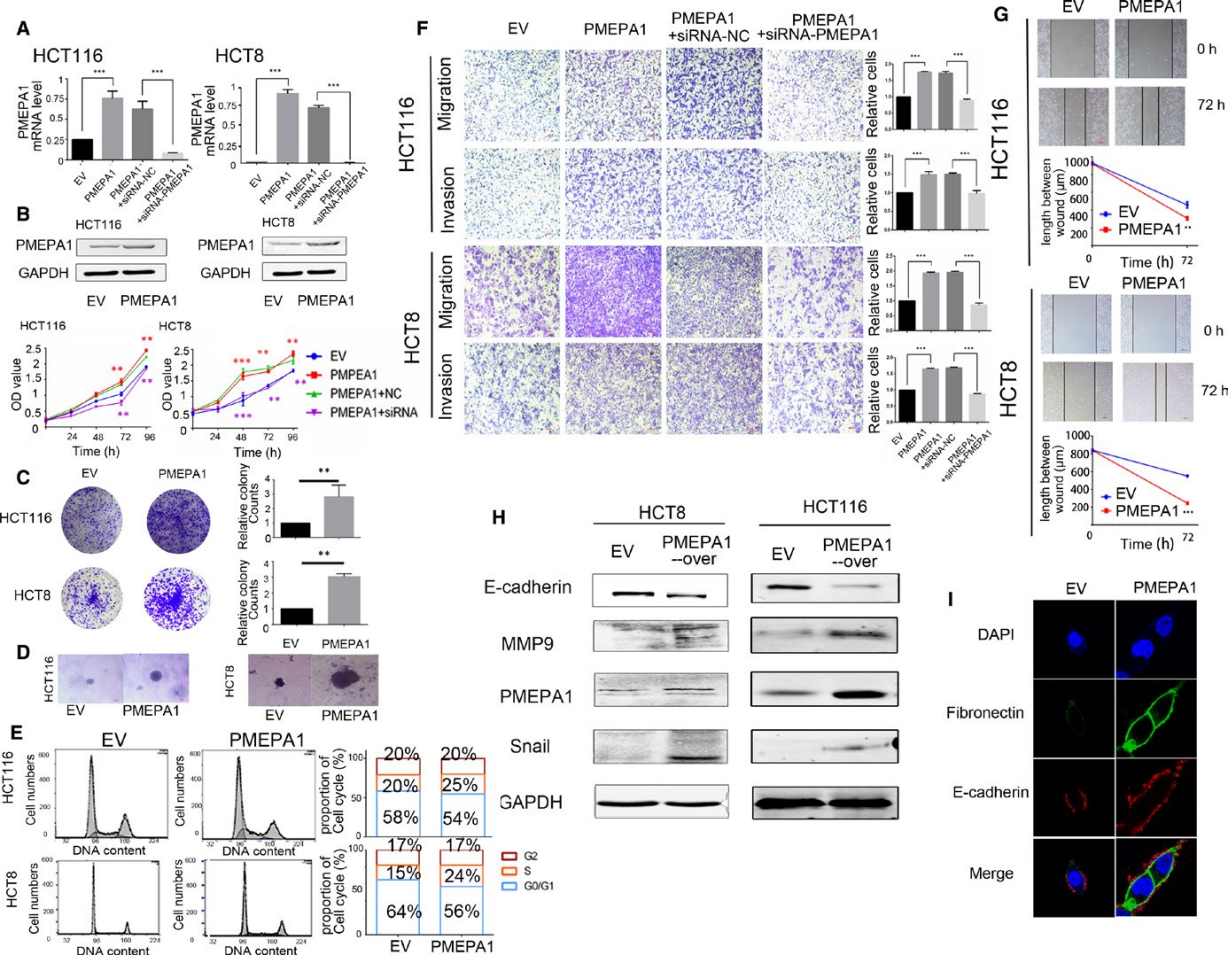
Prostate transmembrane protein androgen induced 1 (PMEPA1) promotes proliferation and metastasis in colorectal cancer cell lines (A) The efficiency of overexpressed PMEPA1 has been testified by qRT‐PCR and Western blot in HCT116 and HCT8. And the efficiency of siRNA‐PMEPA1 has been testified by qRT‐PCR. B, The proliferation of HCT116 and HCT8 cells with PMEPA1 overexpressed and PMEPA1‐overexpressed+siRNA‐PMEPA1 was detected by CCK8 assay. The * markers in purple colours indicate the statistical analysis is between PMEPA1+NC and PMEPA1+siRNA; the * markers in red colours indicate the statistical analysis is between EV and PMEPA1. (C,D) The proliferation of HCT116 and HCT8 cells with PMEPA1 overexpressed was detected by plate clone assay and soft agar clone assay. E, Flow Cytometer detected the cell cycle and the proportion of each cell cycle. F, Migration and invasion assay have been used for HCT116 and HCT8 cells with PMEPA1 overexpressed and PMEPA1‐overexpressed+siRNA‐PMEPA1.The chambers were washed by 30% acetic and the absorbance of washing solution was recorded at 570 mn for the quantification of the relative migration and invasion cells. G, Wound healing assay has been used for the HCT116 and HCT8 cells with PMEPA1 overexpressed. And the length of the wound has been measured by Image J. The designations for levels of significance were used within this figure: *P < 0.05; **P < 0.01; ***P < 0.001; ns, not significant. H, Western blot detection of E‐cadherin, MMP9, snail and PMEPA1 in HCT116 and HCT8 with PMEPA1 up‐regulation. I, Immunofluorescence assay for expression of E‐cadherin and Fibronectin in PMEPA1 up‐regulated in HCT116 cells. The designations for levels of significance were used within this figure: *P < 0.05; **P < 0.01; ***P < 0.001; ns, not significant

As shown in Figure S1A, we chose PMEPA1‐lower‐expressed cell lines, HCT116 and HCT8, to build stable PMEPA1‐overexpressed cell lines (testified shown Figure 3A). Compared with the empty vector (EV), PMEAP1 promoted the proliferation and clones formation (Figure 3B‐D) and decreased the G1/S arrest (Figure 3E). Moreover, PMEPA1 promoted migration and invasion, and speeded of wound healing of CRC (Figure 3F,G). In the HT29 and SW620 cell lines, we also detected the proteins changes of EMT markers. The overexpression of PMEPA1 decreased the expression of E‐cadherin and increased the expression of MMP9 and Snail (Figure 3H). Immunofluorescence assay also validated that PMEPA1 overexpression decreased the expression of E‐cadherin, and increased fibronectin in HCT116 cells lines (Figure 3I). In order to confirm that the changed phenotypes are related to PMEPA1, we transfected siRNA‐PMEPA1 and Negative control (siRNA‐NC) into PMEPA1‐overexpressed cell lines. As shown in Figure 3B, PMEPA1‐siRNA inhibited the proliferation compared with siRNA‐NC in PMEPA1 stable expressing HCT116 and HCT8 cell lines. Transwell migration and invasion assays also indicated PMEPA‐siRNA inhibited migration and invasion in the PMEPA‐overexpressed cell lines (Figure 3F). As a result, PMEPA1 promotes proliferation via arresting G1/S cell cycle and enhances migration and invasion via EMT in vitro.

### 3.4 PMEPA1 promotes proliferation and metastasis in vivo

**Figure 4:**
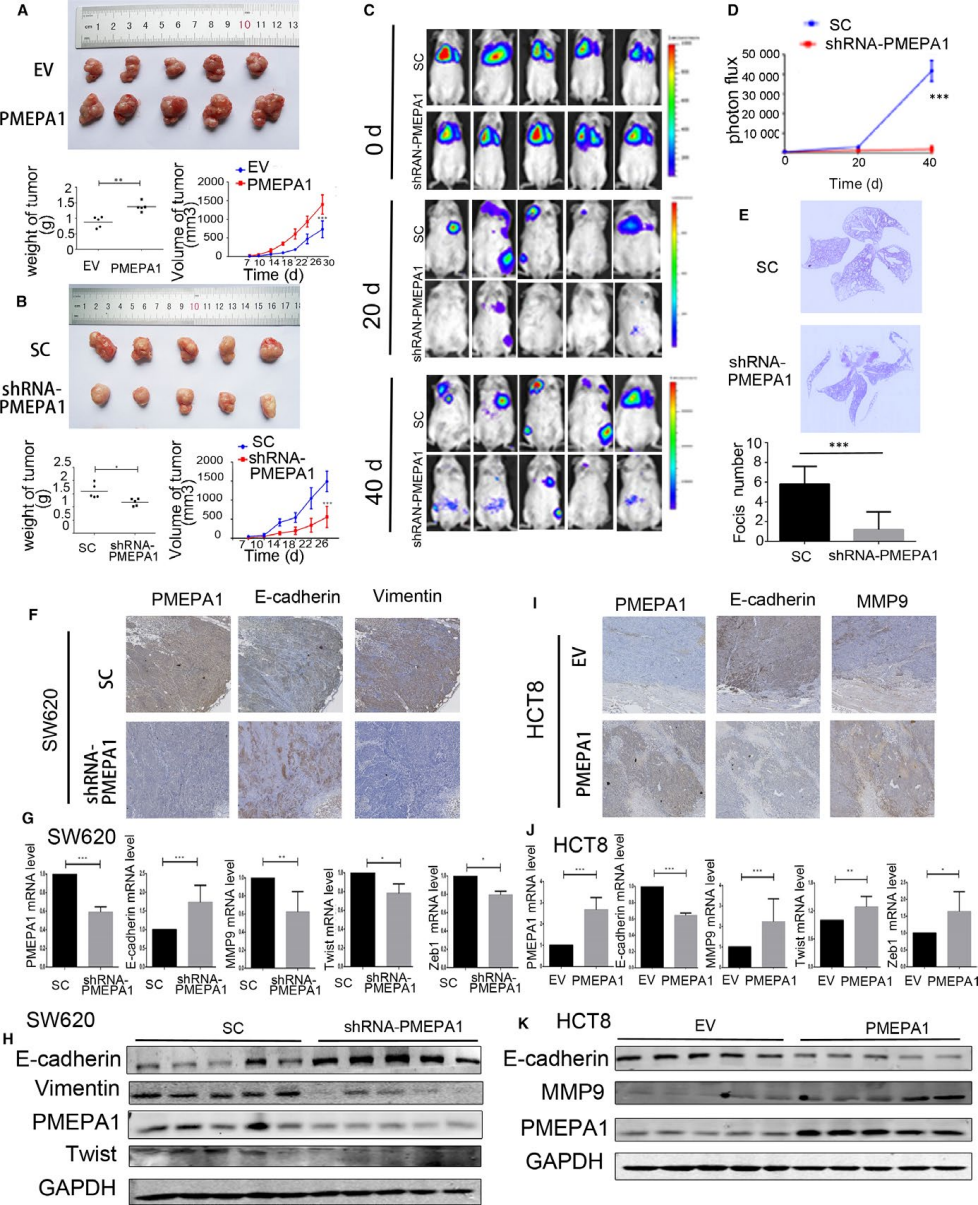
Prostate transmembrane protein androgen induced 1 (PMEPA1) promotes proliferation and metastasis in vivo (A) PMEPA1‐overexpressed HCT8 cells and the control HCT8 cells (EV) have been subcutaneously inoculated into immune‐deficient mice. And the volume of xenografts was recorded after injection. After 30 d, the weight of xenografts has been photographed and measured after killing the mice. B, PMEPA1‐knockdown SW620 cells and control SW620 cells have been subcutaneously inoculated into immune‐deficient mice. And the volume of xenografts was recorded after injection. After 26 d, the weight of xenografts has been photographed and measured after killing the mice. C, Representative images of luciferase signals and (D) quantification of photon flux for metastasis by tail‐vein injection of SW620‐SC and SW620‐shRNA PMEPA1 cells in immune‐deficient mice. E, H&E staining for pulmonary metastatic foci from SW620‐SC and SW620‐shRNA PMEPA1cells. And the foci in lungs of SW620‐SC and SW620‐shRNA PMEPA1 groups were counted. F, Immunohistochemistry detection for PMEPA1, E‐cadherin, Vimentin in SW620‐SC and SW620‐shRNA PMEPA1 xenografts. G, qRTPCR dectection for PMEPA1, E‐cadherin, MMP9, Twist and Zeb1vimentin in SW620‐SC and SW620‐shRNA PMEPA1 xenografts. H, Western blot detection for E‐cadherin, vimentin, PMEPA1 and Twist in SW620‐SC and SW620‐shRNA PMEPA1 xenografts. I, Immunohistochemistry detection for PMEPA1, E‐cadherin, MMP9in HCT8‐EV and HCT8‐PMEPA1 xenografts. J, qRT‐PCR dectection for PMEPA1, E‐cadherin, MMP9, Twist and Zeb1vimentin in HCT8‐EV and HCT8‐PMEPA1 xenografts. (K) Western blot detection for E‐cadherin, vimentin and PMEPA1 in HCT8‐EV and HCT8‐PMEPA1 xenografts. The designations for levels of significance were used within this figure: *P < 0.05; **P < 0.01; ***P < 0.001; ns, not significant

To validate the role PMEPA1 in vivo, we subcutaneously inoculated control and PMEPA1‐overexpressed HCT8 cells into male BALB/c nude mice. The continuous tumour volume measurement indicated the average of tumour volume of the PMEPA1‐overexpressed group is larger than the control group. The weight of xenografts with PMEPA1 overexpressed were higher than the control xenografts which also confirms the proliferation induction of PMEPA1 (Figure 4A). Furthermore, we inoculated control and PMEPA1‐knockdown SW620 cell into male BALB/c nude mice, the continuous tumour volume measurement and the weight of the tumour indicated PMEPA1 knockdown reduces the proliferation in vivo (Figure 4B).

We then intravenously injected luciferase‐labelled control and PMEPA1 knockdown SW620 cells into NOD/SCID mice and monitored metastasis with bioluminescent imaging. After 40 days, the whole‐body luminescence signals in the PMEPA1 knockdown group were ~10‐fold lower than the control group (Figure 4C,D). Moreover, the number of metastatic foci in the lung decreased significantly in the mice which were injected with PMEPA1 knockdown cells (Figure 4E). The results above indicate that PMEPA1 knockdown decreases the metastasis in mice.

Expression of E‐cadherin was increased and the expression of Vimentin was decreased in the PMEPA1‐knockdown xenografts (Figure 4F). However, in the PMEPA1‐overexpressed xenograft, expression of E‐cadherin was decreased and expression of MMP9 was increased (Figure 4I).

We then detected the mRNA levels of PMEPA1, E‐cadherin, MMP9, Twist and Zeb1 of the xenograft. E‐cadherin was decreased and MMP9, Twist and Zeb1 were increased in the PMEPA1‐knockdown xenografts (Figure 4G). And E‐cadherin was increased and MMP9, Twist and Zeb1 were decreased in PMEPA1‐overexpressed xenografts (Figure 4J). In the PMEPA1‐knockdown group, protein levels protein E‐cadherin was increased and the expression of Twist and Vimentin were decreased (Figure 4H). And protein levels of E‐cadherin were decreased in the PMEPA1‐overexpressed xenografts; and the protein levels of MMP9 were decreased in the PMEAPA1‐knockdown group (Figure 4K).

Moreover, we performed a correlation analysis between the expression of PMEPA1 and EMT markers. The results indicated a strong positive correlation between PMEPA1 and CHD2, Twist, FN1. However, the result indicated PMEPA1 was negatively correlated with CDH1 (E‐cadherin) (Figure S1B). Taken together, PMEPA1 promoted CRC proliferation, EMT and metastasis in vivo.

### 3.5 PMEPA1 has dual role in TGF‐β signalling

To explore the mechanism that PMEPA1 promotes EMT and metastasis. We analysed the public data from TCGA and GSE35834, and screened the gene expression profiles associated with PMEPA1 in CRC samples. Then GSEA enrichment analysis was performed in these different expressed genes (Figure S2). TGF‐β signalling pathway has been enriched from the gene positively regulated by PMEPA1. And the metastasis related pathways, Focal adhesion, ECM‐receptor interaction and regulation of actin cytoskeleton were enriched. Moreover, oncogenic pathways like pathways in cancer and Wnt signalling pathways were also enriched.

**Figure 5:**
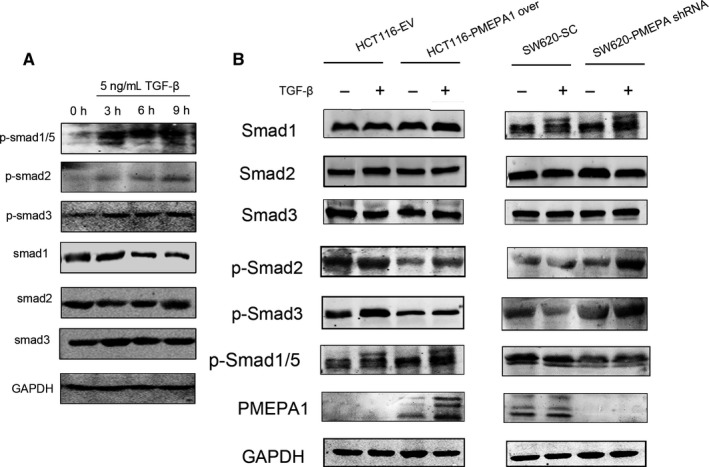
Prostate transmembrane protein androgen induced 1 (PMEPA1) regulates non‐TGF‐β signalling (A) Under different treatment duration of 5 ng/mL TGF‐β, the expressions of Smad 1, 2, 3 and phosphorylation level of Smad2, Smad3 and Smad1/5 in HCT116 cells. B, The expressions of Smad 1, 2, 3 and phosphorylation level of Smad2, Smad3 and Smad1/5 in HCT116 cells with PMEPA1 overexpressed and SW620 cells with PMEPA1 knocked‐down with TGF‐β treatment

As we know, the role of PMEPA1 is complicated in TGF‐β signalling. We then explored the relationship between PMEPA1 and TGF‐Β signalling in CRC. We then tested phosphorylation of smad1/5, smad2 and smad3 after 3, 6 and 9 hours treated with 5 ng/mL TGF‐β to find the optimal point of time when phosphorylation was activated (Figure 5A). As shown in Figure 5B, we tested the changes of downstream proteins of canonical TGF‐β signalling in PMEPA1 knockdown cells and PMEPA1‐overexpressed cells and its corresponding control cells after 6 hours treated with TGF‐β. We found PMEPA1 has inhibited the phosphorylation of Smad2 and smad3 which indicated PMEPA1 blocked the canonical TGF‐β signalling. Then we explored the role of PMEPA1 on BMP signalling, the non‐canonical TGF‐β signalling. Interestingly, in the PMEPA1‐overexpressed cells the phosphorylated smad1/5 was increased. In the PMEPA1 knockdown cells, this phosphorylated smad1/5 was decreased. We also tested several downstream genes in the BMP signalling pathways, and with the increase of PMEPA1, the expressions of ID1, ID2, ID3 and smad6 were up‐regulated in HCT116 cell line (Figure S1D). Taken together, PMEPA1 inhibits the canonical TGF‐β signalling, but activates the BMP signalling.

To explore the role of BMP signalling in EMT induced by PMEPA1, we first verified whether PMEPA1 interacts with Smad1, a significant protein in BMP signalling. As shown in Figure S1C, the co‐immunoprecipitation assay indicated no interaction between and Smad1. Moreover, to explore the role of BMP signalling pathway, we used siRNA‐smad1 to decrease the expression of smad1 in the PMEPA1‐overexpressed cells (Figure S1E,F). And the decreased mRNA level of E‐cadherin caused by overexpressed PMEAP1 has been rescued by the siRNA‐smad1 (Figure S1G). As shown in Figure S1H, the decreased Smad1 inhibited the cell mobility induced by the PMEPA1. Taken together, PMEPA1 promotes migration via BMP signalling pathways.

## 4 DISCUSSION

Prostate transmembrane protein androgen induced 1 (PMEPA1) is highly expressed in most of cancers, like lung cancer, breast cancer and prostate cancer.^9^, ^17^, ^19^, ^20^, ^21^, ^33^ And we found that the expression of PMEPA1 was significantly up‐regulated in CRC samples of the TCGA and GEO databases, which indicated PMEPA1 might play an oncogenic roles in CRC. More importantly, the high PMEPA1 level in tumour samples was correlated with the poor survival of CRC patients, which indicated that PMEPA1 could be considered as an independent prognostic marker.

However, some studies showed PMEPA1 could inhibit tumour proliferation and metastasis. In prostate cancer, androgen receptor signalling is a significant pathway for the cancer progression. However PMEPA1 accelerates NEDD‐meditated derogation of androgen and inhibits the androgen receptor signalling, moreover, PMEPA1 inhibits the prostate cancer cell proliferation.^11^, ^34^ PMEPA1 also suppresses prostate cancer metastases to bone by inhibition of TGF‐β signalling and interfering the formation of complex of Smad2/3 and Smad4.^13^ In this study, the experiments data in vivo and in vitro showed PMEPA1 promoted migration and invasion as a pro‐metastatic molecule. We speculated that the tissue or organ specific expression of PMEPA1 might cause the contradictory phenotype in colorectal cancer. In some types of cancer, PMEPA1 has been reported to promote cancer metastasis. In breast cancer, PMEPA1 reduces PTEN and promotes non‐canonical PI3K/Akt signalling to promote cancer progression.^33^ In prostate cancer, PMEPA1 inhibits Smad3/4–cmyc–p21Cip1 signalling pathway to promote prostate cancer cell proliferation.^10^ In lung cancer, PMEPA1 regulates ROS and IRS‐1 signalling and induces EMT to promote metastasis.^35^ Together, PMEPA1 could be considered as a versatile molecule, which functions the complex roles in different types of cancer.

As we know, TGF‐β is a classical inducer of EMT to drive tumour cell migration, invasion, and metastasis in many carcinomas.^36^ As a TGF‐β‐inducible gene, PMEPA1 regulated multiple biological process in several types of cancer.^16^, ^20^, ^37^ Some reports showed PMEPA1 accelerates the metastasis and EMT through TGF‐β signalling.^35^ Nevertheless, we demonstrated PMEPA1 promoted migration and invasion, and induced EMT in CRC by activating the BMP signalling pathway with phosphorylation of Smad1 and Smad5. Previous reports have indicated the role of BMP signalling pathways in EMT. In colorectal cancer, BMP‐2, an inducer of BMP signalling, induces EMT and drug resistance.^38^ BMP‐2 also induces BMP signalling and EMT in pancreatic cancer.^39^ The agonists of BMP‐2 and BMP‐7 also block the BMP signal, and inhibit EMT and invasion in melanoma cells.^40^ PMEPA1, which activates BMP signalling pathway like BMP‐2 and BMP7, might have the potential to be a novel target for molecular agonists design. However, the detailed mechanism of PMEPA1 activating BMP signalling remained to further study.

In conclusion, we revealed that PMEPA1 promoted EMT‐mediated metastasis through activating TGF‐β non‐canonical signalling cascade. Although the conclusion needs more clinical studies to valid, PMEPA1 might has the potential to serve as a meaningful biomarker for high‐risk CRC or to serve as a therapeutic target for intervene colorectal cancer.

## CONFLICT OF INTEREST

We declare that we have no financial and personal relationships with other people or organizations that can inappropriately influence our work, there is no professional or other personal interest of any nature or kind in any product, service and/or company that could be construed as influencing the position presented in, or the review of, the manuscript entitled.

## AUTHORS’ CONTRIBUTION

Xue Wang analysed the data has shown in Figure 1 and Tables 1, 2, Lei Zhang and Xue Wang have contributed in Figures 2, 3, 4, 5, and Figure S1, S2. Lei Zhang and Xue Wang have contributed in the manuscript writing. All authors have participated in editing and reviewed the manuscript.
