# Supplementary figures and images for: PMEPA1 induces EMT via a non‐canonical TGF‐β signalling in colorectal cancer
Source: J Cell Mol Med. 2019 Mar 19;23(5):3603–15. doi: 10.1111/jcmm.14261 (PMC6484414; doi:10.1111/jcmm.14261)

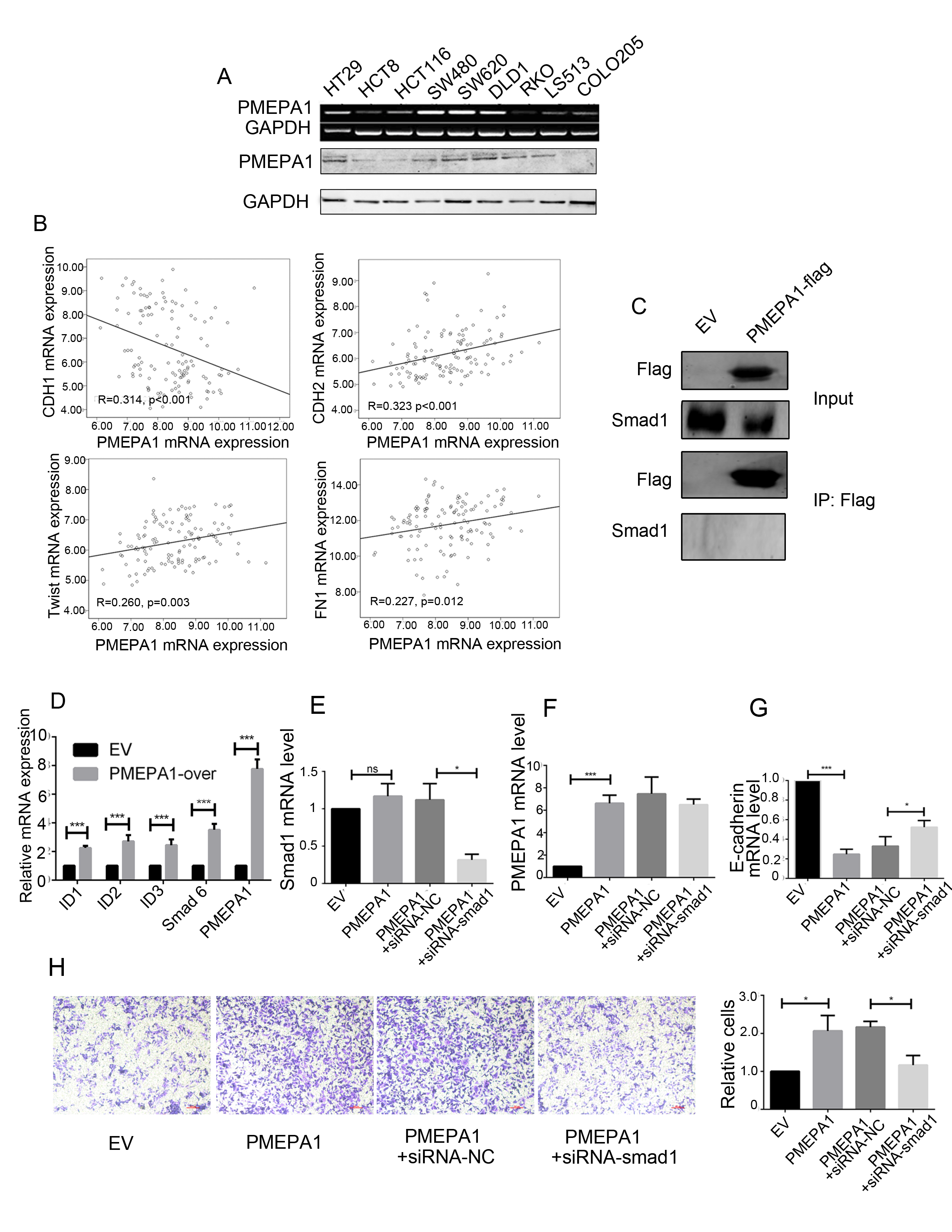

Supplement: Supplementary file 1 [file JCMM-23-3603-s001.tif]

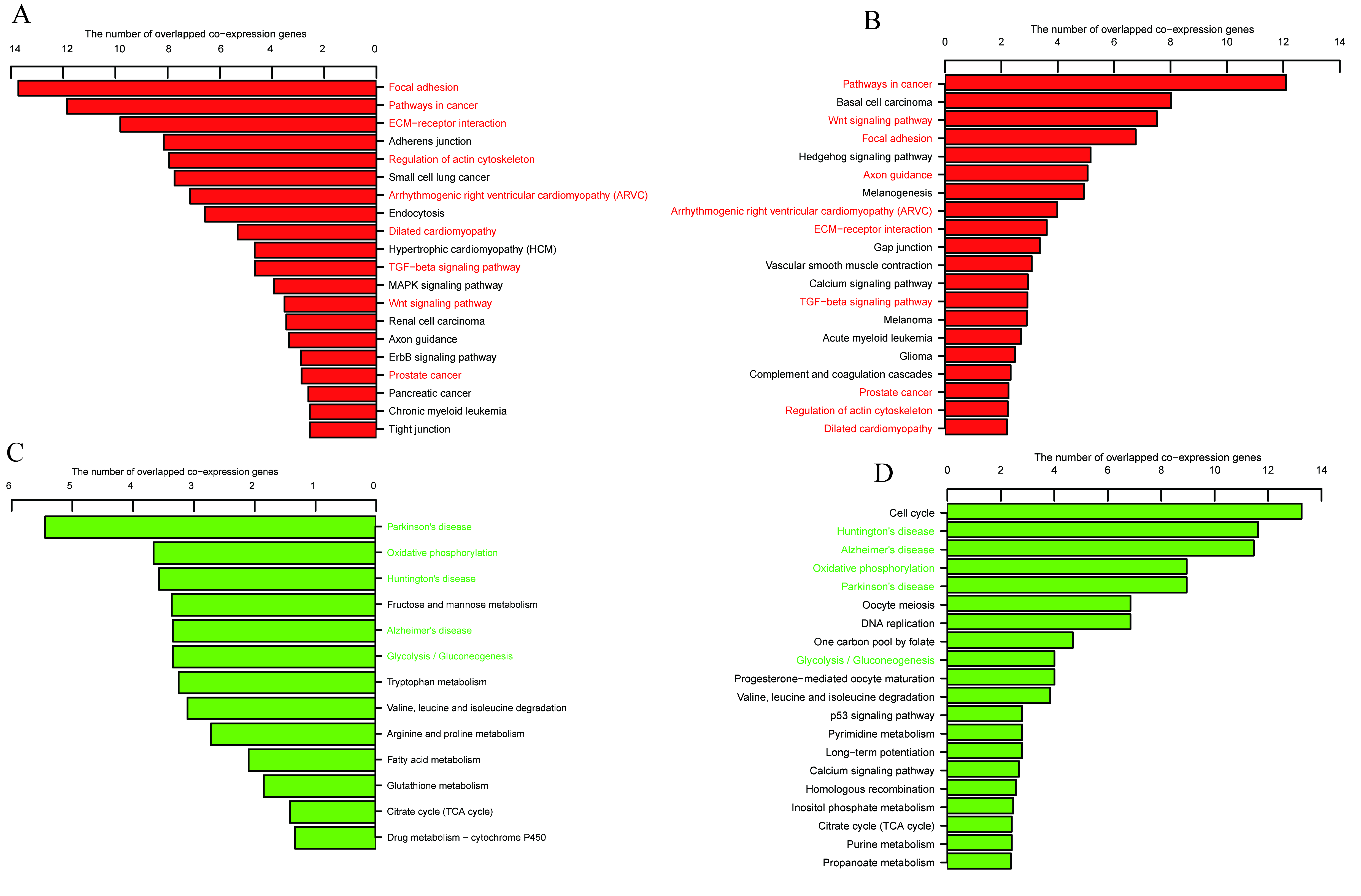

Supplement: Supplementary file 2 [file JCMM-23-3603-s002.tif]
